# Community Intervention to Promote Consumption of Fruits and Vegetables, Smoke-free Homes, and Physical Activity Among Home Caregivers in Bogotá, Colombia

**Published:** 2006-09-15

**Authors:** Diego I Lucumí, Olga L Sarmiento, Roberto Forero, Luis F Gomez, Gladys Espinosa

**Affiliations:** Health División, Fundación FES Social; School of Medicine, Universidad de los Andes, Bogotá, Colombia, and Centro de Estudios e Información en Salud, Fundación Santa Fe de Bogotá; Simpson Center for Health Services Research, University of New South Wales, Sydney, Australia; Health División, Fundación FES Social, Bogotá, Colombia; Secretaría Distrital de Salud de Bogotá, DC

## Abstract

**Introduction:**

We conducted a pilot study to develop and assess the effectiveness of three interventions to promote consumption of fruits and vegetables, promote physical activity, and negotiate smoke-free homes among home caregivers in Bogotá, Colombia. Colombian home caregivers were defined as women who take care of minors in their local communities regardless of kinship or family ties.

**Methods:**

A nonrandomized community intervention was conducted in low socioeconomic status neighborhoods in Bogotá. Ninety-seven women aged 18 to 60 years participated in one of three groups. In groups A and B, women received the following components: information and communication about healthy behaviors (with group A receiving additional activities); education about developing decision-making skills; and social support from family members and others. In group C, women received only the information and communication component received by group B. The main outcomes (measured at baseline, immediately after the intervention at 5 months, and at 7 months) included self-reported consumption of fruits and vegetables, whether there was an agreement form signed by family members to refrain from smoking inside the home, and self-reported level of physical activity.

**Results:**

No differences were found between intervention groups. Regardless of the intervention, there was an increase in the proportion of women who reported consuming juices made from fruit (from 51.5% at baseline to 80.9% at 7 months, *P* <.001), an increase in the proportion of women who reported daily consumption of vegetables or salad (from 44.1% at baseline to 64.7% at 7 months, *P* < .001), and an increase in the proportion of homes with an agreement that forbids in-home smoking (from 27.9% at baseline to 44.1% at 7 months, *P* = .04). There was no significant difference in levels of physical activity from baseline to postintervention.

**Conclusion:**

Home caregivers may be responsive to community interventions associated with the promotion of healthy diet and agreements with family members who smoke to refrain from smoking in the home.

## Introduction

Latin American countries are experiencing an epidemic of chronic diseases that are having an overwhelmingly detrimental impact on the health of their populations (1,2). In Colombia, the leading cause of mortality is cardiovascular disease (121 deaths per 100,000 population) (3).

Evidence shows that unhealthy diets, smoking, and physical inactivity are risk factors for noncommunicable diseases, such as cardiovascular disease, cancer, and diabetes (4,5). In Bogotá, the capital of Colombia, low consumption of fruits and vegetables, tobacco use, and physical inactivity constitute a growing public health concern (6). 

Women from this city are reported to be less physically active than men according to the Centers for Disease Control and Prevention's (CDC's) recommendations (61.9% of women are physically inactive compared with 56.2% of men) (7); also, men are more likely to smoke (38.6% of men compared with 19.1% of women). No sex differences are apparent in the consumption of fruits and vegetables, with an overall mean (± SD) consumption of servings per day of 2.5 (± 2.2) (6). Another study in Bogotá as shown that 44% of women reported two or more lifestyle risk factors such as low consumption of fruits and vegetables, sedentary lifestyle, smoking, or alcohol consumption (8).

In the last decade, some interventions have focused on changing dietary behaviors (9), reducing exposure to environmental tobacco smoke (10), and promoting physical activity (11). In Latin America and the Caribbean, the Pan American Health Organization developed *Conjunto de acciones para la reducción multifactorial de las enfermedades no transmisibles (CARMEN — *Initiative for Integrated Noncommunicable Disease Prevention), a program that promotes research and interventions to prevent chronic disease (2). In this context, previous authors have pointed out that community interventions must be fitted to the social and cultural characteristics of the region (11,12).

Women have been identified as a priority group for public health interventions related to chronic diseases (13,14). Women from impoverished families support each other by looking after not only their own children but also children of family members or neighbors. They may play a critical role in improving healthy behaviors in their communities by becoming role models for the adoption of health behaviors (14). Also, these women have been recognized as potential leaders and may have standing in their local communities (15). 

We hypothesized that home caregivers with the appropriate community intervention could increase fruit and vegetable consumption, be able to negotiate smoking cessation with family members who smoke, and increase their own levels of physical activity. To evaluate this hypothesis, we developed three interventions related to the district program *Vive mejor, aliméntate sanamente, libérate del humo, ejercítate con frecuencia y sé feliz (Tu Vales)* (Live better, eat healthy, be free of smoke, exercise frequently, and be happy [You Are Valuable]).

To assess the effectiveness of these three interventions and to learn about logistical problems before initiating a large-scale intervention, we conducted a pilot study in four neighborhoods of one locality of Bogotá

## Methods

### Theoretical framework

Social cognitive theory was used to guide the intervention because of its usefulness in assessing personal and environmental factors associated with chronic diseases (16). The underlying concept of social cognitive theory is that human behaviors are explained in terms of a triadic, dynamic, and reciprocal model in which behaviors, personal factors, and environmental factors interact (16). These concepts are described in Table 1.

To guide our community intervention, we created a model comprised of three levels: individuals, neighborhoods, and localities (Figure). In this article, we focus on the individual level. The other levels will be explored in future analyses.

**Figure 1:**
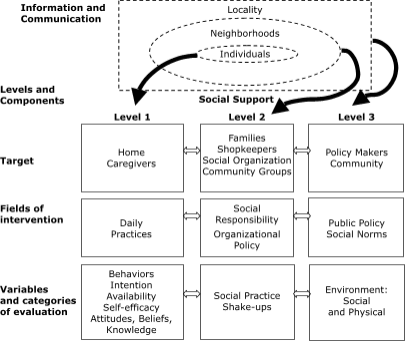
Community model to promote consumption of fruits and vegetables, smoke-free environments, and physical activity among home caregivers, Bogotá, Colombia, 2003.

**Table:** 

This diagram is a community model to promote consumption of fruits and vegetables, smoke-free environments, and physical activity among home caregivers in Bogota, Colombia. It reads from top to bottom, left to right, respectively.
Category titles are listed in bold down the first column. They include: “Information and Communication,” “Levels and Components,” “Target,” “Field of intervention,” and “Variables and categories of evaluation.”
The next column begins by listing “Individuals” in the Information and Communication stage. From there, the column leads to “Level 1” and continues listing “Home Caregivers,” “Daily Practices,” and “Behaviors, Intention, Availability, Self-efficacy, Attitudes, Beliefs, Knowledge.”
The third column begins by listing “Neighborhoods” in the Information and Communication stage. From there, the column leads to “Level 2” and continues listing “Families, Shopkeepers, Social Organization, Community Groups,” “Social Responsibility, Organizational Policy,” and “Social Practice Shake-ups.”
The final column begins by listing “Locality” in the Information and Communication stage. From there, the column leads to “Level 3” and continues listing “Policy Makers, Community,” “Public Policy, Social Norms,” and “Environment: Social and Physical.”

### Study setting

Bogotá is located at 8650 feet above sea level. The year-round temperature is between 7°C and 18°C (with no seasonal changes). *Tu Vales* was conducted in four neighborhoods of Santa Fe, one of 20 localities of Bogottá. Santa Fe has a population of 107,044; 70% of this population is of low socioeconomic status (SES) (17). Santa Fe was selected for this study because it was targeted in 2000 as a demonstration area for developing chronic disease prevention programs. In addition, this locality had the highest prevalence rate of cardiovascular disease in Bogotá in 1999 (18).

The four neighborhoods in Santa Fe were selected because they were prioritized by the government to receive social programs, and community organizations in each neighborhood agreed to promote the participation of home caregivers.

### Study population

For this study, a caregiver was defined as any woman aged 18 to 60 years who had lived in Santa Fe for 1 year or more and who had looked after children younger than 15 years for at least 12 months in her residence or local neighborhood. Women who reported mental or serious physical disabilities were excluded from the study. Community leaders invited approximately 35 women identified as home caregivers from each neighborhood. Approximately 120 of them attended the first information session. Of these, 20 could not participate because of time constraints.

Home caregivers received information about the intervention and signed a written consent form. Women who agreed to participate received an apron and a T-shirt as incentives. In addition, at the end of the intervention and evaluation, women and their families were invited to a formal closing ceremony with the research team and the mayor of the locality and received a certificate for their participation. The study was approved by the institutional ethics committee of the Fundación FES Social.

### Study design

This study was a nonrandomized community-based intervention including three phases: development, implementation, and evaluation. The pilot study was conducted from January 2003 to February 2004. To design the materials for the information and communication and education components, we used social marketing strategies (products, price, place promotion) (19). These strategies took into account daily life practices and cultural, social, and economic characteristics of the target population.

### Implementation

The study comprised three groups: A (intervention group plus), B (intervention group), and C (partial intervention). These groups were geographically separated from one another. The intervention consisted of three components: information and communication, education, and social support.

The information and communication component explored participants' knowledge about nutrition, smoking, and physical activity. The education component emphasized skill development and autonomy to make healthy decisions. The social support component involved community members such as family, peers, and owners of grocery stores. 

Components were delivered according to each intervention group and behavioral intervention as shown in Table 2. All three groups received the information and communication component. For this component, participants took part in 16 weekly sessions (2 hours each) delivered in each of the community rooms. Physical activity and smoke-free home sessions were provided together in eight sessions, and fruits and vegetables activities were presented in the other eight sessions. A nutritionist (for fruits and vegetables sessions), social worker (for smoke-free home sessions), and physiotherapist (for physical activity sessions) conducted these activities.

**Fruits and vegetables intervention**

As part of the information and communication component, women received printed information about the benefits of eating fruits and vegetables. Women were also informed about places where and seasons during which they could buy fresh fruits and vegetables at low prices. In addition, women received recipes that incorporate fruits and vegetables so that they could increase their dietary intake. As part of the education component, women were able to adapt and create their own recipes. Groups A and B also received a social support component, which consisted of participation of family members or peers during group sessions and a grocery store intervention. Grocery store owners were visited by a nutritionist and invited to participate in a session where they receive information about their role in promoting healthy eating behaviors in their communities and discuss strategies to promote consumption of fruits and vegetables, such as reducing the price and establishing in-store campaigns to educate consumers about which fruits and vegetables are in season.

**Smoke-free home intervention**

As part of the information and communication component, women received printed information about the deleterious effect of first- and second-hand smoking. Communication activities included group discussions and the development of a skit relating family experiences with tobacco consumption. The education component involved developing strategies to empower participants to obtain written agreements with smokers in the family to restrict smoking inside their homes. Women from group A received three additional home visits to reinforce the importance of the written agreement to not smoke in enclosed areas at home.

**Physical activity intervention**

As part of the information and communication component, all home caregivers received informative materials about the benefits of being physically active. Additionally, women received informational materials about how to maximize their physical activity and how to use the places and opportunities of their daily routines to be physically active. Communication activities included group discussions about enablers of and barriers to being physically active. Only women from group A were contacted three times by telephone to complete a questionnaire identifying barriers to physical activity and assessing changes in their stages of physical activity as defined by the transtheoretical model and stages of change theory proposed by Proschaska et al (20). After barriers and stages were identified, the educator provided guidelines for overcoming barriers and moving to the next stage. As part of the educational component, all women participated in physical activities including stretching, dance classes, and toning exercises.  Additionally, women learned how to measure their heart rate. Women from groups A and B participated in brisk walking in their neighborhood (e.g., in parks, on footpaths). As part of the social support component, women from groups A and B identified key peers (family members or friends), who were invited to participate in the educational activities.

### Quantitative evaluation

During face-to-face interviews, all women completed a questionnaire on three separate occasions: before beginning the intervention (baseline), immediately after the intervention (at 5 months), and 2 months after the intervention (at 7 months). Interviewers were trained by the principal investigators, and the data collection process was supervised to identify and prevent interviewer bias.

The primary outcome for dietary habits was based on assessments of daily intake of fruits and vegetables using the following questions:

Do you drink juices made of fruits every day?

Not counting juices made of fruits, do you eat whole fruit every day?

Do you eat vegetables or salads every day?

These questions had been culturally adapted in a previous study in Santa Fe (6) and took into account the nutrition module of the Non-Communicable Disease Surveillance Toolkit designed by the Pan American Health Organization (21).

The outcome for smoke-free environment was a written agreement between the family member who smoked and the home caregiver and a verbal confirmation to the researchers about fulfillment of the agreement. 

The primary outcome for physical activity was self-reported participation in regular exercise. These outcome measures were obtained from questions of the Colombian Spanish version of the short format of the International Physical Activity Questionnaire (IPAQ) (22). For walking, women were classified into the following patterns: *regular pattern* (those who reported walking for at least 150 minutes in bouts of at least 10 minutes for at least 5 days during the last week) and *irregular pattern* (those who reported walking less than 150 minutes in bouts of at least 10 minutes for at least 5 days during the last week).

Physical activity during leisure time was dichotomized (regular vs irregular or inactive). Women who had a regular pattern of leisure-time physical activity were those who reported moderate leisure-time physical activity for at least 30 minutes per day in cumulative bouts of at least 10 minutes each for 5 or more days per week *or* who reported vigorous leisure-time physical activity for at least 20 minutes per session for 3 or more days per week. Women who had an irregular pattern or who were inactive were those who reported participating in moderate or vigorous physical activity for at least 10 minutes at a time but who did not comply with all aspects of the regular activity category.

### Statistical analysis 

Our analytic strategy involved three steps. First, we compared sociodemographic characteristics between baseline and postintervention populations of the intervention groups using the Χ^2^ test. Second, we assessed intervention effects across groups by using generalized estimating equations (GEE); we used Proc Genmod (SAS Institute Inc, Cary, NC) because outcome variables were dichotomous. GEE model procedures allowed us to control for clustering of women according to neighborhoods. For each multivariate analysis model, the outcome corresponded to the status of fruit and vegetable consumption, nonsmoking agreement, or physical activity pattern postintervention. The independent variable was neighborhood group (A, B vs C) as a dummy coded variable and baseline measurement of each behavior. We only adjusted for baseline measure because of small sample size. In addition, possible covariates that could have been main confounders are sex and SES, but the study population was homogeneous with respect to these factors. Analyses were conducted on complete cases at 7 months after the intervention (n = 70). Third, we compared outcome proportions according to time of evaluation (baseline vs 5 months and baseline vs 7 months). In this pilot study, the analysis was not done on an intention-to-treat basis because of missing data on outcome variables and high variability of women participating across educational programs. All statistical analyses were conducted with SAS software version 8.0 (SAS Institute Inc, Cary, NC).

### Qualitative evaluation

After obtaining the results from the statistical analysis, the researchers organized a focus group to obtain insight about the intervention, present the results to the women who had participated, and assess their perceptions about their behavioral patterns. Participants watched a video developed by the research team about their participation in the project and the graph results with the main outcomes. The focus group moderator asked them 1) their opinion about the results that had been shown; 2) what they had learned from the intervention; and 3) what could be done to improve outcomes. Two researchers transcribed the answers given by participants and coded and sorted them according to relevant behavioral categories, such as outcomes and lessons learned, and suggested ways to improve the interventions.

## Results

### Quantitative results

A total of 97 women who satisfied the inclusion criteria participated in the study (24 in group A, 45 in group B, and 28 in group C). Seventy-two percent of the women who were interviewed at baseline completed the three surveys. Table 3 shows the sociodemographic characteristics of all participants at baseline and those who completed the three interview surveys. The only significant difference between those who completed the three surveys and those who completed only the baseline survey was affiliation with community groups (*P* = .03) (Table 3).

At baseline, approximately half of the women reported consuming fruits every day (51.5% for fruit juices and 54.5% for whole fruit) (Table 4). At baseline, 44.1 % reported consuming vegetables or salads every day. There was a significant increase in the proportion of women who reported consuming juices made from fruit, from 51.5% at baseline to 80.9% at 7 months (*P *<.001) (Table 4).

Table 5 shows changes from baseline to postintervention for each intervention group, and Table 6 shows the comparison of group outcomes at 7 months. No statistically significant differences were found between intervention and partial intervention groups (Table 6). However, there was a greater increase in the consumption of fruit juices of group C from baseline to 7 months compared with groups A and B (Table 5). In all groups there was also a significant increase in the daily consumption of vegetables or salads from 44.1% at baseline to 66.2% at 5 months and 64.7% at 7 months (Table 4). The consumption of fresh fruits showed a statistically nonsignificant pattern at baseline. Among all groups at baseline, 13.4% of women reported that people smoke inside their homes (Table 4). This proportion decreased to 6.0% at 5 months and 9% at 7 months. Additionally, the proportion of women reporting the existence of a smoke-free home agreement increased from 27.9% at baseline to 39.7% at 5 months and to 44.1% at 7 months (Table 4). However, the proportion of women who reported a smoke-free agreement at 7 months was lower in group A than in groups B and C (Table 5).

At baseline, 19.7% of the women in all groups reported walking at least 150 minutes per week and 33.8% reported engaging in physical activity during leisure time (Table 4). The proportion of women meeting CDC recommendations for physical activity showed a nonsignificant increase from baseline to 5 months and from baseline to 7 months. There were no statistically significant differences between intervention and partial intervention groups for physical activity outcomes (Table 6).

### Qualitative evaluation 

During the focus group, 13 women reported that the intervention helped them to identify new and easy ways to prepare fruit and vegetable recipes. They also highlighted the benefits of the social interaction with other women during the workshops. In addition, the workshops allowed them to share different types of fruit and vegetable recipes. They also expressed that purchasing seasonal produce was less expensive than out-of-season produce. Participants also reported redistributing their family budget to increase fruit consumption. Despite strategies to lower the amount of money spent on fruits, women perceived that fresh fruit was more expensive than juice and therefore decided that fruit juices were a better alternative for the family. 

Women recognized that the intervention gave them evidence about the negative effects of first- and second-hand smoke, evidence that was useful in the negotiation and fulfillment of the nonsmoking agreement. However, they found that these agreements could take a long time to establish.**

Participants reported that the intervention increased their awareness about the importance of physical activity, especially walking, in daily life. They also recognized that physical activity had different dimensions. They reported that unsafe areas in their communities were barriers to physical activity.

Finally, they expressed being happy with their participation in *Tu Vales* and were motivated to participate in this kind of intervention in the future.

## Discussion

*Tu Vales *is one of the first community-based interventions in Latin America with a multicomponent approach to promote consumption of fruits and vegetables, smoke-free home environments, and physical activity. We consider this pilot study to have been useful in exploring a community-based model to promote healthy behaviors adapted to our socioeconomic and cultural context.

The results provided some evidence that interventions with home caregivers that provided information, communication, and education could increase or maintain postintervention changes for overall increases in the consumption of fruits and vegetables and in the establishment of smoke-free home agreements. The qualitative results provided insight about the context of social practice, perceptions, empowerment, and knowledge. These results have important implications for the development of future interventions among women in these communities.

Although we found a significant difference in affiliation with community groups between women completing the baseline survey and women completing the postintervention survey, we cannot extrapolate the effects of this variable to a more general model. Future studies should consider these effects and take into account the association between social support, social cohesion, and health behaviors.

### Fruits and vegetables

The changes in the consumption of fruits were mainly due to increased consumption of juices rather than whole fruit. This change could be explained by the fact that fruit juice is less expensive than whole fruit. Home caregivers reported that cost was a barrier to the consumption of fruits, especially for large families (23,24). In contrast, costs were not a major barrier for vegetable consumption, and the overall increase in vegetable consumption also could be associated with the wide availability of some vegetables that are both popular and traditionally accepted. 

Another important finding is that home caregivers recognized the importance of informative and educational strategies to learn about availability of seasonal fruits and family budget distribution. In this context, our findings could guide interventions in future programs such as the proposed program *Bogotá in hambre* (Bogotá without Hunger) (25).

This study provides important evidence for recommending that policy makers and local government agencies increase the availability, quality, and variety of fruits and vegetables in grocery stores and supermarkets in low SES neighborhoods. In addition, we consider that engaging shopkeepers and primary producers at the local level in future interventions could have an important impact in promoting consumption of fruits and vegetables at the community level.

### Smoke-free homes 

Considering the complexity of the social context around smoking, our intervention, which focused on family support, needs to be reinforced with social and legal norms (9). As a first step, this project produced an encouraging increase in the adoption of smoke-free agreements and also demonstrated that home caregivers can successfully negotiate these agreements. In addition, this agreement could contribute to smoke-free homes because during the negotiation, women and their families may increase their knowledge about the effects of smoke and second-hand smoke, and they can provide support to smokers contemplating quitting (9).

### Physical activity

In *Tu Vales* we did not find significant differences in walking behaviors after the interventions. The lack of significant differences could be related to the fact that we did not distinguish between walking for other purposes and walking for recreation. Nonetheless, we need to continue developing interventions to promote walking among poor caregivers in Latin America because walking is the most prevalent physical activity among women (26,27).

Although we did not find differences across groups in physical activity behaviors, we found that after the educational intervention to increase awareness of the recreational facilities in their neighborhoods, women were more likely to identify recreational facilities of low cost or no cost in their neighborhoods for their physical activities. More research is needed to demonstrate that home caregivers can be role models for physical activity behavioral interventions because of their influence on children under their care, relatives, and friends.

### Limitations and conclusion

This study had several limitations. Our findings should be taken with caution because of the groups' small sample size, number of participants lost to follow-up, short time of the intervention, and use of some questions, such as the ones used to evaluate the consumption of fruits and vegetables, that did not measure serving sizes.

Nevertheless, the results of this study could be useful to identify enablers of and barriers to consumption of fruits and vegetables, smoke-free homes, and regular physical activity and contribute to the establishment of information, communication, and education methodologies to carry out community-based interventions among poor women. Some lessons learned from this study are related to the potential of involving home caregivers in community interventions, the necessity of establishing strategies that address the physical and social environment, the usefulness of qualitative and quantitative methods, and the advantages of using public health theories and models to conduct and evaluate community-based interventions in a similar context.

As reported in the qualitative results, some of these interventions, such as the implementation of the smoke-free home agreement, may require a long time for dissemination and implementation. However, the framework of *Tu Vales* will continue to be used to explore and develop phase 2 and phase 3 interventions in Bogotá and other metropolitan areas nationally. We hope this type of intervention can also be used in other developing countries with similar sociocultural characteristics. The next phase of *Tu Vales* is a large-scale intervention with study populations to include home caregivers, shopkeepers, and leaders of community groups in 32 neighborhoods.

## Figures and Tables

**Table 1:** Concepts in Social Cognitive Theory Used to Guide *Tu Vales* Intervention for Home Caregivers, Bogotá Colombia, 2003

**Concept**	**Implications for Intervention**
Environment	Participation of families and shopkeepers to provide social support
Situation	Correction of misperceptions about consumption of fruits and vegetables, exposure to smoke, and participation in physical activity
Behavioral capability	Activities to educate participants about selecting, buying, and preparing fruits and vegetables; promoting smoke-free homes; and engaging in daily physical activity
Observational learning	Learning skills developed during group sessions from teacher or other caregivers
Reciprocal determinism	Promotion of behaviors among home caregivers, intervention on the environment (families, peers, and shopkeepers)

**Table 2:** Components of *Tu Vales* Intervention to Promote Consumption of Fruits and Vegetables, Smoke-Free Homes, and Physical Activity for Three Groups of Home Caregivers, Bogotá Colombia, 2003

**Component**	**Group**		

	**A**	**B**	**C**
**Information and communication**			
Group sessions	Yes	Yes	Yes
Phone counseling for physical activity	Yes	No	No
Home visits to promote smoke-free homes	Yes	No	No
**Education**			
Group sessions	Yes	Yes	Yes
Awareness of places for physical activity	Yes	Yes	No
**Social support**			
Participation of family or peers during group sessions	Yes	Yes	No
Grocery store intervention	Yes	Yes	No

**Table 3:** Sociodemographic Characteristics of Participants in *Tu Vales* Intervention, Bogotá Colombia, 2003^a^

**Characteristic**	**Baseline^b^ (N = 97)**	**Postintervention^c^ (n = 70)**	***P* Value^d^**
Age, mean (SD), y	37.3 (10.5)	38.4 (10.85)	.94
Marital status			
Single	18 (18.6)	10 (14.3)	.77
Married	28 (28.9)	23 (32.9)	
Living with partner	36 (37.1)	24 (34.3)	
Separated or widowed	15 (15.5)	13 (18.6)	
No. of children living in household, mean (SD)	2.1 (1.6)	2.1 (1.6)	.99
Education, y			
1-5	27 (28.4)	20 (29.0)	.91
6-11	54 (56.8)	40 (58.0)	
12-23	14 (14.7)	9 (13.0)	
Principal activity during last 30 days			
Working	58 (59.8)	44 (62.9)	.64
Working/studying	4 (4.1)	3 (4.3)	
Looking for job	4 (4.1)	2 (2.9)	
Housewife	31 (32.0)	21 (30.0)	
Paid work			
Yes	61 (62.9)	47 (67.1)	.41
No	36 (37.1)	23 (32.9)	
Household salary per month, U.S. $			
<128	83 (85.6)	59 (84.3)	.48
≥128	14 (14.4)	11 (15.7)	
Community mother			
Yes	19 (19.6)	17 (24.3)	.32
No	78 (80.4)	53 (75.7)	
Affiliation with community groups			
Yes	22 (31.4)	10 (14.7)	.03
No	48 (68.6)	58 (85.3)	
Places where you buy most of food for household^e^			
Grocery store	23 (38.3)	19 (40.4)	.91
Market square	13 (21.7)	7 (14.9)	
Supermarket	23 (38.3)	21 (44.7)	
Other	1 (1.7)	0 (0)	
Health insurance			
Yes	79 (81.4)	57 (81.4)	.99
No	18 (18.6)	13 (18.6)	
Perceived health status			
Excellent or very good	11 (11.3)	6 (8.6)	.18
Good	55 (56.7)	44 (62.9)	
Poor or average	31 (32.0)	20 (28.6)	
Body mass index, kg/m^2^, mean (SD)	27.7 (5.5)	27.8 (5.6)	.99
Intervention groups			
A	24 (24.7)	18 (25.7)	.99
B	45 (46.4)	31 (44.3)	
C	28 (28.9)	21 (30.0)	

a Values are no. (%) unless otherwise indicated.

b No. of participants in a category may not sum to 97 because of missing responses.

c No. of participants in a category may not sum to 70 because of missing responses.

d Differences between proportions or means at baseline and proportions or means at postintervention (7 months from baseline) determined by Cochran Q test of homogeneity.

e This question answered only by women who buy groceries for the household.

**Table 4:** Changes in Health-related Outcome Measures from Baseline to Postintervention Among Participants (n = 68), *Tu Vales* Intervention, Bogotá Colombia, 2003

**Outcome Measure**	**Baseline, %**	**5 months, %**	***P* a**	**7 months, %**	***P* a**
**Fruits and vegetables**					
Consume fruit juice daily	51.5	58.8	.24	80.9	<.001
Consume whole fruit daily	54.5	69.6	.06	55.9	.87
Consume vegetables or salad daily	44.1	66.2	<.001	64.7	<.001
**Smoke-free home**					
People smoke inside home^b^	13.4	6.0	.06	9.0	.32
Agreement exists restricting smoking in home	27.9	39.7	.08	44.1	.04
**Physical activity**					
Walk for transportation or recreation at least 150 min/wk^c^	19.7	27.3	.17	19.7	.99
Engage in physical activity during leisure time	33.8	38.2	.58	48.5	.09
Perceive that recreational facilities of low or no cost exist in neighborhood	55.9	72.1	.02	66.2	.16
Report that it is easy to use recreational facilities on Saturdays and holidays^b^	77.6	70.2	.30	71.6	.39

a Differences between baseline and immediately after intervention (5 months) and between baseline and 7 months determined by Χ^2^ test.

b Response missing for one participant.

c Response missing for two participants.

**Table 5:** Changes from Baseline to Postintervention in Health-related Outcome Measures Among Participants in *Tu Vales* Intervention, by Intervention Group, Bogotá Colombia

**Outcome Measure**	**Group A (n = 18)**			**Group B (n = 29)**			**Group C (n = 21)**		

	**Baseline, %**	**Post-intervention, %**		**Baseline, %**	**Post-intervention, %**		**Baseline, %**	**Post-intervention, %**	
		
		**5 mos.**	**7 mos.**		**5 mos.**	**7 mos.**		**5 mos.**	**7 mos.**
**Fruits and vegetables**									
Consume fruit juice daily	38.9	50.0	77.8	62.1	48.3	75.9	47.6	81.0	90.5
Consume whole fruit daily	27.8	77.8	66.7	62.1	62.1	55.2	66.7	71.4	47.6
Consume vegetables or salads daily	33.3	55.6	55.6	41.4	58.6	62.1	57.1	85.7	76.2
**Smoke-free home**									
People smoke inside home	16.7	0	11.1	21.4	14.3	10.7	0	0	4.8
Agreement exists restricting smoking in home	22.2	22.2	27.8	37.9	55.2	51.7	19.1	33.3	47.6
**Physical activity**									
Walk for transportation or recreation at least 150 min/wk	27.8	50.0	33.3	24.1	20.7	20.7	5.3	15.8	5.3
Engage in physical activity during leisure time	72.2	38.9	55.6	34.5	48.3	51.7	0	23.8	38.1
Perceive that recreational facilities of low or no cost exist in neighborhood	66.7	77.8	77.8	44.8	75.9	58.6	61.9	61.9	66.7
Report that it is easy to use recreational facilities on Saturday and holidays	94.1	70.6	82.4	72.4	72.4	65.5	71.4	66.7	71.4

**Table 6:** Comparison of Intervention Groups for Health-related Outcomes at Postintervention, *Tu Vales* Intervention, Bogotá Colombia, 2003

**Outcome**	**Group A Compared With Group C**		**Group B Compared With Group C**	

	**Odds Ratio^a^ (95% CI)**	***P^b^* Value**	**Odds Ratio^a^ (95% CI)**	***P* Value**
**Fruits and vegetables**				
Consume fruit juice daily	0.3 (0.03-2.71)	.29	0.2 (0.03-1.53)	.12
Consume whole fruit daily	1.8 (0.25-12.35	.56	1.0 (0.13-7.01)	.97
Consume vegetables or salads daily	0.4 (0.04-3.62)	.40	0.4 (0.05-3.36)	.41
**Smoke-free home**				
People smoke inside home	1.0 (0.02-40.74)	.99	2.3 (0.06-80.29)	.65
Agreement exists restricting smoking in home	0.5 (0.04-5.18)	.54	1.5 (0.15-15.72)	.71
**Physical activity**				
Walk for transportation or recreation at least 150 min/wk	4.2 (0.34-52.41)	.26	1.4 (0.12-16.50)	.80
Engage in physical activity during leisure time	2.4 (0.16-34.81)	.52	2.4 (0.23-25.28)	.46
Perceive that recreational facilities of low or no cost exist in neighborhood	1.9 (0.21-17.34)	.56	1.4 (0.21-9.91)	.71
Report that it is easy to use recreational facilities on Saturdays and holidays	1.3 (0.08-19.44)	.87	1.0 (0.12-7.91)	.99

CI indicates confidence interval.

a Odds ratios adjusted at baseline value to compare groups; C is reference group.

b *P* values for odds ratios are from the generalized estimating equations.
